# Camptothecin, triptolide, and apoptosis inducer kit have differential effects on mitochondria in colorectal carcinoma cells

**DOI:** 10.1002/2211-5463.13401

**Published:** 2022-04-14

**Authors:** Veronika Liskova, Marek Kajsik, Barbora Chovancova, Ladislav Roller, Olga Krizanova

**Affiliations:** ^1^ Institute of Clinical and Translational Research Biomedical Research Center Slovak Academy of Sciences Bratislava Slovakia; ^2^ Department of Chemistry Faculty of Natural Sciences University of Ss. Cyril and Methodius Trnava Slovakia; ^3^ Institute of Zoology Slovak Academy of Sciences Bratislava Slovakia

**Keywords:** apoptosis inducer kit, camptothecin, colorectal carcinoma, fission, mitochondria, triptolide

## Abstract

Mitochondrial fission and fusion are required for cell survival, and several studies have shown an imbalance between fission and fusion in cancer. High levels of mitochondrial fusion are observed in drug‐resistant tumor cells, whereas mitochondrial fission may be important in sensitizing tumor cells to chemotherapy drugs. Based on current knowledge, we hypothesized that different chemotherapeutics might differentially affect mitochondrial dynamics and energy production. Thus, we selected chemotherapeutics with different mechanisms of action (camptothecin, triptolide and apoptosis inducer kit) and investigated their effect on mitochondria in colorectal carcinoma cells. We report that these chemotherapeutics decreased the activity of complex I and reduced the mitochondrial membrane potential, and also decreased the size of mitochondria in the colorectal carcinoma cell lines DLD1 and HCT‐116. Treatment with camptothecin, triptolide and/or apoptosis inducer kit results in differential effects of fission on apoptosis in these cells. Our results suggest that fission is an important process in apoptosis induced by chemotherapeutics.

## Graphical abstract

Mitochondria constantly change their morphology according to the requirements of the cell. In cancer cell lines (DLD1 and HCT‐116), we observed a decrease in the size of mitochondria compared to a non‐cancer cell line EAhy926. Treatment with camptothecin, triptolide, and/or apoptosis inducer kit decreased the activity of complex I, increased reactive oxygen species, reduced mitochondrial membrane potential and induced apoptosis in cancer cells.

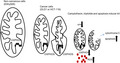

**Abbreviations**
AIK: apoptosis inducer kit
ANOVA: analysis of variance
Drp1: dynamin‐related protein 1
Mfn: mitofusin
ntRNA: non‐targeting RNA
OPA1: optic atrophy 1 protein
PBS: phosphate‐buffered saline
ROS: reactive oxygen species
TTL: triptolide

In the majority of cells, mitochondria play an irreplaceable role in ATP production. These organelles not only function as a powerful source of energy and substrates required for a surplus of cell functions, but also participate in the regulation of cell viability, differentiation, and apoptosis [1]. Nevertheless, mitochondria are also deeply involved in modulation of a process of tumorigenesis because they affect cancer initiation, growth, survival, and metastasis formation. Multiple aspects of mitochondrial biology beyond bioenergetics support transformation, including mitochondrial biogenesis and turnover, fission and fusion dynamics, cell death susceptibility, oxidative stress regulation, metabolism, and signaling [2].

Mitochondrial function is highly dependent on two processes that significantly affect mitochondrial morphology: fission and fusion. Mitochondrial fission is dependent on the activity of a highly conserved dynamin‐related GTPase, Drp1. Mitochondrial fusion is a more complex process, where both outer and inner mitochondrial membrane fusion must be realized. Mitofusins 1 (Mfn1) and 2 (Mfn2), which are embedded on the mitochondrial outer membrane, are responsible for outer membrane fusion. When the process of outer membrane fusion is accomplished, another dynamin family member, Optic atrophy 1 protein (OPA1), mediates inner membrane fusion [3]. Mitochondrial fission and fusion are continual processes, and they are required for survival, with the aim of adapting to changes in the surrounding environment. In cancer, several studies have shown an imbalance between fission and fusion processes. In cancer cells, elevated fission activity or decreased fusion is responsible for fragmented mitochondrial network. In variety of cancer cell types, elevated Drp1 expression is connected with a migratory phenotype, which highlighted the importance of mitochondrial dynamics in the processes of metastasis formation [4]. When Drp1 knockdown/inhibition or Mfn2 overexpression is applied, cancer cell growth is impaired, thus suggesting the importance of mitochondrial network remodeling in tumorigenesis. In neuroblastoma cells, mitochondrial protein rearrangement and mitochondrial elongation are responsible for resistance to treatment induced by cisplatin. Inhibition of mitochondrial fusion as a result of Mfn1 silencing increases cisplatin sensitivity [5]. Moreover, in lung adenocarcinoma cells, overexpression of OPA 1 protein enhanced cisplatin resistance via inactivation of apoptosis dependent on caspase activity [6]. Nevertheless, when the process of mitochondrial fragmentation is inhibited, it efficiently blocked the release of cytochrome *c* and subsequent cell death [7]. These and other studies suggest that fission and fusion are important modulators of a fate of tumor cells. A high level of mitochondrial fusion always exists in resistant tumor cells, whereas mitochondrial fission may be important in sensitizing tumor cells to chemotherapy drugs.

Mitochondria are also considered as major producers of reactive oxygen species (ROS). Excessive ROS production caused by dysfunction of the oxidative phosphorylation may result (or participate) in the development of several diseases. In cancer cells, increased ROS production was detected, which promotes cancer development by the induction of variety of processes (e.g. genomic instability, modulation of a gene expression, and participation in different signaling pathways). ROS is involved in the process of proliferation and the accompanying upregulation of antioxidant pathways prevents ROS‐mediated cytotoxicity [8, 9]. On the other hand, ROS overproduction was shown to augment cisplatin‐induced apoptosis [10].

Based on current knowledge, we hypothesized that different chemotherapeutics might differently affect mitochondrial dynamics and energy production. Thus, we selected two chemotherapeutics with different mechanisms of action, as well as a mixture of chemotherapeutics, and investigated their effect on mitochondria with the impact of fission on apoptosis induction in colorectal carcinoma cells.

## Materials and methods

### Cell cultivation and treatment

For experiments, human colon adenocarcinoma cell line DLD1 (ATCC, CCL‐221), endothelial EAhy926 cell line (ATCC, CRL‐2922TM), and human colon carcinoma cell line HCT‐116 (ATCC, CCL‐247) were cultured in Dulbecco’s minimal essential medium (Sigma‐Aldrich, St. Louis, MO, USA) or RPMI medium (Sigma‐Aldrich) with a high glucose (4.5 g·L^−1^) and l‐glutamine (300 μg·mL^−1^), supplemented with 10% fetal bovine serum (Sigma‐Aldrich), penicillin (Calbiochem, San Diego, CA, USA; 100 U·mL^−1^), and streptomycin (Calbiochem; 100 μg·mL^−1^). Cells were cultured in a water‐saturated atmosphere at 37 °C and 5% CO_2_ as described previously [11]. In some groups, apoptosis was induced by an apoptosis inducer kit (AIK) (1 µL·mL^−1^). The AIK (Calbiochem, catalog. no. 178486) is composed of five ready‐to‐use chemical reagents that induce apoptosis through different mechanisms: actinomycin D (inhibits RNA synthesis), camptothecin (an inhibitor of nuclear topoisomerase), cycloheximide (an inhibitor of protein synthesis), dexamethasone (induces apoptosis probably by binding and activating the intracellular glucocorticoid receptor), and etoposide (inhibits topoisomerase activity). In other groups, camptothecin (2 µm), or triptolide (TTL; 100 nm), which affects mainly ATP‐dependent DNA helicase XPB, was used. The concentration of camptothecin used was the same as that in AIK. The concentration of triptolide was selected from a calibration curve from our previous experiments [12] and verified by IC_50_ determination. Cells were incubated with these compounds for 24 h before being subjected to further analysis.

### Cell viability and IC_50_ determination

Cell viability was determined using the redox indicator AquaBluer™ (MultiTarget Pharmaceuticals, Colorado Springs, CO, USA). Viable cells turn AquaBluer™ from oxidized (non‐fluorescent blue) form to the reduced (fluorescent red) form. The fluorescence intensity of AquaBluer™ corresponds to the number of viable cells in the sample. The assay was carried out in accordance with the manufacturer’s recommendations. Cells at a concentration of 7 × 10^3^ were plated on 96‐well plate 24 h prior to treatment. After 24 h of treatment, 100 μL of diluted AquaBluer™ (1 : 100) was added. After 4 h of loading at 37 °C 5% CO_2_ in the dark, fluorescence was measured using a fluorescence scanner Synergy H1 Reader (BioTek, Bad Friedrichshall, GE, USA) at an excitation of 540 nm and an emission of 590 nm. Viability was calculated as: %Viability = (RFU_test_/RFU_veh_) × 100, where RFU_veh_ is the average relative fluorescence units of the no‐drug wells.

### Detection of apoptosis with Annexin V‐FLUOS

Cells were gently removed by accutase (Biosera, Kansas City, MO, USA) pelleted at 1000 ***g*** for 5 min and then washed with 1 mL of 1 × phosphate‐buffered saline (1× PBS, pH 7.4). Cell pellet was resuspended in solution containing 50 μL of 1× Binding Buffer (BioVision, San Francisco, CA, USA) and 2 μL of Annexin V‐FLUOS (Roche Diagnostics, Indianapolis, IN, USA) and incubated at room temperature in the dark for 20 min in accordance with the manufacturer’s instructions. After incubation, 200 μL of 1 × Binding Buffer and 5 μL of 7‐amino‐actinomycin D Viability Staining Solution (Thermo Fisher Scientific, Waltham, MA, USA) was added; samples were placed on ice and measured on CytoFLEX S flow cytometer (Beckman Coulter, Brea, CA, USA).

### Determination of the size of mitochondria

Cells were grown on glass coverslips (amount 3 × 10^4^) pretreated with polylysine (Sigma‐Aldrich). Afterwards, cells were treated with MitoTracker Red CMXRos (dilution 1 : 2000; Thermo Fisher Scientific) for 30 min at 37 °C and 5% CO_2_ in the dark. Next, coverslips were washed in serum‐free medium, washed in 1 × PBS, and then fixed in ice‐cold methanol. Then, cells were rehydrated in 1 × PBS and the coverslips were mounted onto slides in mounting medium with 4′,6‐diamidino‐2‐phenylindole dihydrochloride (Sigma‐Aldrich). Cells were visualized by confocal microscopy using Leica TCS SPE II with upright DM5500 microscope and 405 and 532 nm excitation lasers (Leica Microsystems, Mannheim, Germany). las af software (Leica Microsystems) was used for acquisition of images and measurement of the length of mitochondria.

### MitoTracker Green FM

Cells were plated on a 24‐well plate at the density of 1 × 10^5^. After 24 h of treatment, cells were washed with serum‐free medium and loaded with MitoTracker Green FM probe (Thermo Fisher Scientific) at a final concentration of 100 nm in a well for 30 min at 37 °C and 5% CO_2_ in the dark. Afterward, cells were washed twice with 1 × PBS. Fluorescence was measured using a Synergy H1 Reader (BioTek) at an excitation of 490 nm and an emission of 516 nm.

### Determination of oxidative stress

Cellular oxidative stress was determined by measuring of ROS using CellROX® Orange Reagent (Thermo Fisher Scientific) as described previously [13]. Cells were plated on a 24‐well plate at the density of 2 × 10^5^. As a positive control, pyocyanine was used (50 µm for 4 h; Sigma‐Aldrich). After treatment, cells were washed with serum‐free medium and incubated with CellROX® Orange Reagent at a final concentration of 5 µm in a well for 30 min at 37 °C and 5% CO_2_ in the dark. Next, cells were washed with 500 µL of 1 × PBS. Fluorescence was measured using a Synergy H1 Reader (BioTek) at an excitation of 545 nm and an emission of 565 nm. The results were expressed as arbitrary units of fluorescence.

### Cytofluorometric analysis of the mitochondrial membrane potential (Ψm)

Changes in mitochondrial membrane potential (Ψm) were performed as described previously [14]. Briefly, cells were collected by centrifugation at 1000 ***g*** for 5 min and washed with 1 × PBS. Incubation was performed in 200 μL of PBS/0.2% BSA containing 4 μm·L^−1^ 5,5′,6,6′‐tetrachloro‐1,1′,3,3′‐tetraethylben‐zimidazolyl‐carbocyanine iodide fluorescent dye (JC‐1; Thermo Fisher Scientific) for 25 min at 37 °C in the dark. Cell data were acquired using a BD FACSCanto II flow cytometer (Becton Dickinson, Ann Arbor, MI, USA). JC‐1 green and JC‐1 red were determined. Forward and side light‐scattering characteristics were used to exclude cell debris from the analysis. For each analysis, 1 × 10^5^ cells were acquired, and the ratio of JC‐1 red/JC‐1 green fluorescence of viable cells was used to calculate the decrease in Ψm. Data were analyzed using flowing, version 2.5.1. (https://en.freedownloadmanager.org/Windows‐PC/Flowing‐Software‐FREE.html).

### Gene silencing

Cells were plated on a six‐well plate at a density of 2.5 × 10^5^. For silencing of the gene for DrpI, SMART POOL siRNA from Dharmacon (catalog. no. L‐012092‐00‐0005; Thermo Fisher Scientific) and Dharmafect transfection reagent (Thermo Fisher Scientific) were used as described previously [15]. Cells were washed with serum‐free medium and incubated in serum‐free medium containing siRNA (final concentration of 25 nmol) mixed with Dharmafect transfection reagent. After 4 h, medium with 10% fetal bovine serum was added. As a negative control for RNAi experiments, ON‐TARGETplus Non‐targeting Control Pool (Dharmacon, catalog. no. D‐001810‐10‐05; Thermo Fisher Scientific) was used.

### Western blot analysis

Cells were scraped into 10 mm Tris‐HCl, pH 7.5, 1 mm phenylmethyl‐sulfonyl fluoride (Serva, Heidelberg, Germany)with protease inhibitor cocktail tablets (cOmplete EDTA‐free; Roche Diagnostics, Mannheim, Germany) as described previously [11] and centrifuged for 5 min at 3000 ***g*** at 4 °C. The pellet was resuspended in Tris‐buffer containing 50 µmol·L^−1^ Chaps (Sigma‐Aldrich) and then incubated for 30 min at 4 °C. The lysates were centrifuged for 15 min at 10 000 ***g*** at 4 °C. The protein concentration in supernatants was determined using a Modified Lowry Protein Assay Kit (Thermo Fisher Scientific) [16]. The 15–35 µg of protein extract from each sample was separated via SDS/PAGE, and proteins were transferred to a Hybond‐PVDF membrane (Amersham Biosciences, Amersham, UK) using semidry blotting (Owl™; Thermo Fisher Scientific). Membranes were blocked in 5% non‐fat dry milk in Tris‐buffered saline with Tween‐20 (TBS‐T) overnight at 4 °C and then incubated for 1 h with primary antibody β‐actin (dilution 1 : 5000; ab6276; Abcam, Cambridge, UK), or membranes were blocked in 5% non‐fat dry milk/5% BSA in TBS‐T for 1 h at room temperature and then incubated overnight at 4 °C with appropriate primary antibodies: DrpI (dilution 1 : 1000; ab56788; Abcam), phospho‐DrpI Ser616 (dilution 1 : 1000; #4494; Cell Signaling Technology, Danvers, MA, USA), and phospho‐DrpI Ser637 (dilution 1 : 1000; #4867; Cell Signaling Technology). Following washing, membranes were incubated with horse‐radish peroxidase‐linked secondary goat anti‐mouse/anti‐rabbit antibody (dilution 1 : 10 000; ab6789/ab97200; Abcam) 1 h at room temperature. An enhanced chemiluminescence detection system (Luminata Crescendo Western HRP Substrate; Millipore, Burlington, MA, USA) was used to detect bound antibody. Each membrane was digitally captured using image studio on the C‐DiGit Blot Scanner (LI‐COR Biosciences, Lincoln, NE, USA).

### ATP production measurement

An ATP Colorimetric/Fluorometric Assay Kit was used for ATP level determination (BioVision). Measurements were performed in accordance with the manufacturer’s protocol. Cells (1 × 10^6^ in 100 µL of ATP Assay Buffer) were deproteinized using a Deproteinization Sample Preparation Kit (BioVision). For this, 50 µL of sample was used for each determination. Next, 50 µL of reaction mix was added into the cell plate and incubated at room temperature for 30 min, protected from light. Fluorescence was measured using a Synergy H1 Reader (BioTek) at an excitation of 535 nm and an emission of 587 nm. The final results were expressed as nmol per well (50 µL) of sample.

### Complex I activity measurements

Determination of the activity of complex I was performed using a MitoCheck® Complex I Activity Assay Kit (Cayman Chemical, Ann Arbor, MI, USA) in accordance with the manufacturer’s instructions. Absorbance at 340 nm was measured using a Synergy H1 Reader (BioTek). The assay was performed in kinetic read mode at 25 °C and absorbance was monitored every 30 s for 15 min. Complex I activity was determined as a ratio of the rate of sample well and the rate of vehicle control.

### Pyruvate assay

Pyruvate levels were determined by the Pyruvate Colorimetric/Fluorometric Assay Kit (BioVision) in accordance with the manufacturer’s instructions. Fifty microlitre of sample was used for each determination. Next, 50 µL of reaction mix was added into the cell plate and incubated at room temperature for 30 min, protected from light. Fluorescence was measured using a Synergy H1 Reader (BioTek) at an excitation of 535 nm and an emission of 590 nm.

### Statistical analysis

The exact number of parallels and also the number of cultivations for each experiment is indicated as appropriate. Results are presented as the mean ± SEM. Significant differences among groups were determined by one‐way analsysis of variance (ANOVA). For multiple comparisons, an adjusted *t*‐test with *P*‐values corrected by the Bonferroni method was used (Instat; GraphPad Software Inc., San Diego, CA, USA).

## Results

### Determination of IC_50_ of TTL and camptothecin in DLD1 and HCT‐116 cells

**Fig. 1:**
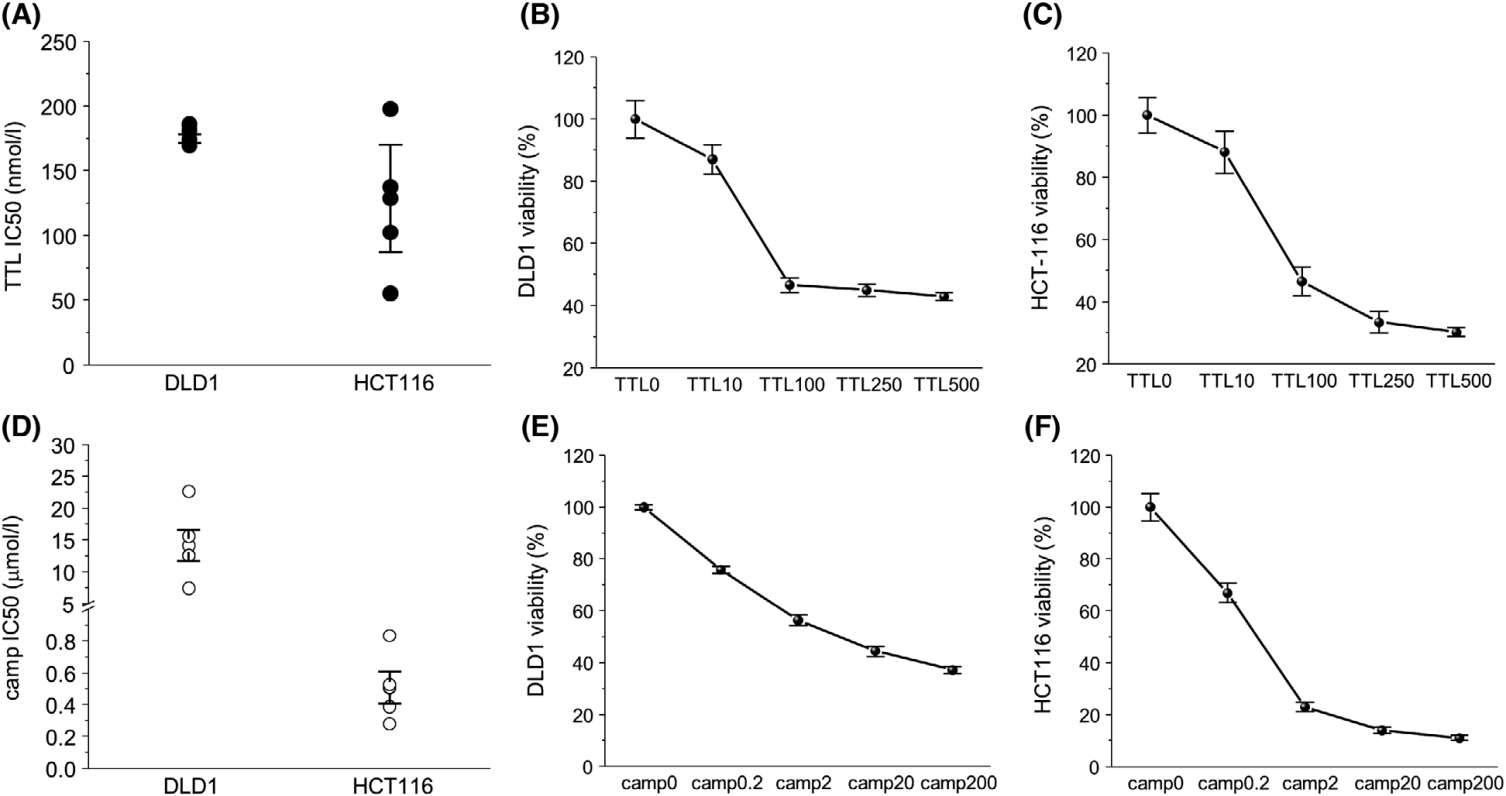
Determination of IC_50_ of TTL and camptothecin (camp) in DLD1 and HCT‐116 cells. IC_50_ of TTL was 179.1 ± 3.2 nm in DLD1 cells and 123.9 ± 41.3 nm in HCT‐116 cells (A). IC_50_ levels of camptothecin significantly differ in DLD1 and HCT‐116 cells, and IC_50_ was 14.14 ± 2.48 µm in DLD1 cells and 0.51 ± 0.1 µm in HCT‐116 cells (D). The results are shown as the mean ± SEM and represent an average of five different cultivations; each cultivation was performed with eight parallels. Typical concentration‐dependent curves are shown in (B) and (C) (for TTL) and (E) and (F) (for camptothecin).

For both, TTL (Fig. 1A) and camptothecin (Fig. 1D), IC_50_ values in DLD1 and HCT‐116 cells were determined. The IC_50_ value of TTL was 179.1 ± 3.2 nm in DLD1 cells and 123.9 ± 41.3 nm in HCT‐116 cells. IC_50_ levels of camptothecin were 14.14 ± 2.48 µm in DLD1 cells and 0.51 ± 0.1 µm in HCT‐116 cells. A typical viability curve for both compounds is shown in Fig. 1B,E for DLD1 cells and in Fig. 1C,F for HCT‐116 cells.

### Changes in apoptosis induction, viability, and levels of ATP and pyruvate in DLD1 and HCT‐116 cells

**Fig. 2:**
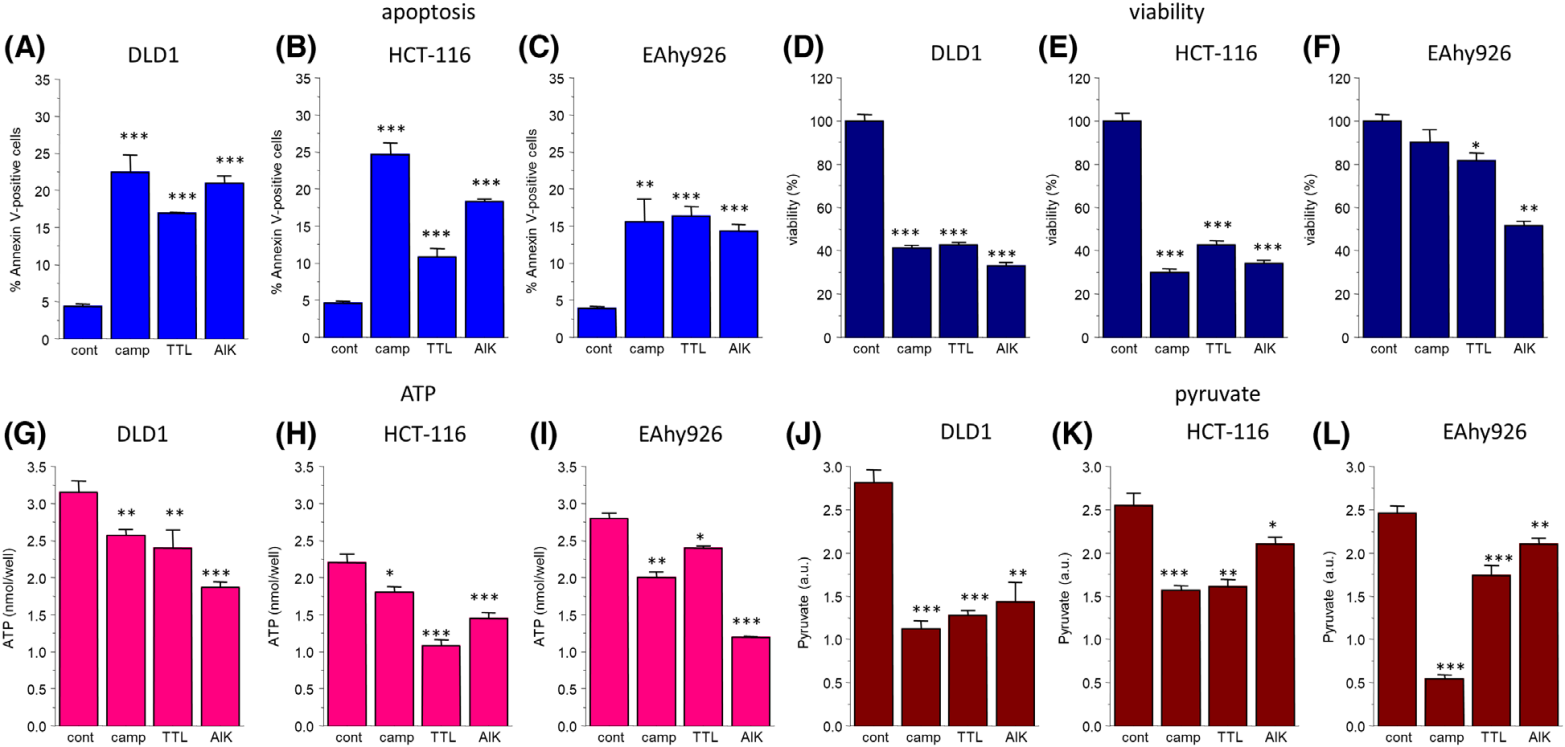
Changes in apoptosis induction (A–C), viability (D–F), and levels of ATP (G–I) and pyruvate (J–L) in DLD1 and HCT‐116 colorectal carcinoma cell lines, as well as the EAhy926 non‐cancer cell line after treatment with camptothecin (camp), triptolide (TTL), and apoptosis inducer kit (AIK). Apoptosis induction was increased in all groups treated with chemotherapeutics (A–C). Accordingly, viability in all treated groups was significantly decreased in cancer cell lines (D, E), but, in the EAhy926 cell line, no significant decrease in the camptothecin‐treated group and only a low decrease in the TTL‐treated group was determined (F). Also, a decrease in ATP levels was detected in all groups treated with chemotherapeutics (G–I) and a reduction was visible also in pyruvate levels (J–L). The results are shown as the mean ± SEM and *n* for each type of experiments (shown as the number of cultivations/parallels in each cultivation) was: apoptosis, *n* = 3/3; viability, *n* = 10/4; ATP, *n* = 3/3; and pyruvate, *n* = 3/3. Significant differences among groups were determined by one‐way ANOVA. For multiple comparisons, an adjusted *t*‐test with *P*‐values corrected by the Bonferroni method was used. *P < 0.05, **P < 0.01, and ***P < 0.001 compared to untreated controls.

All of the chemotherapeutics used (camptothecin, triptolide, and AIK) were able to induce apoptosis in DLD1 (Fig. 2A), HCT‐116 (Fig. 2B), and EAhy926 (Fig. 2C) cells. Apoptosis induction inversely correlates with decreased viability (Fig. 2D,E) in DLD1 and HCT‐116 cells, but, in non‐cancerous EAhy926 cells, the decrease in viability was not so pronounced (Fig. 2F). ATP and pyruvate levels were decreased in all treated groups in both colorectal carcinoma cell lines and also in EAhy926 cells (Fig. 2G–L).

### Activity of complex I, ROS production, and detection of number of cells with decreased mitochondrial membrane potential

**Fig. 3:**
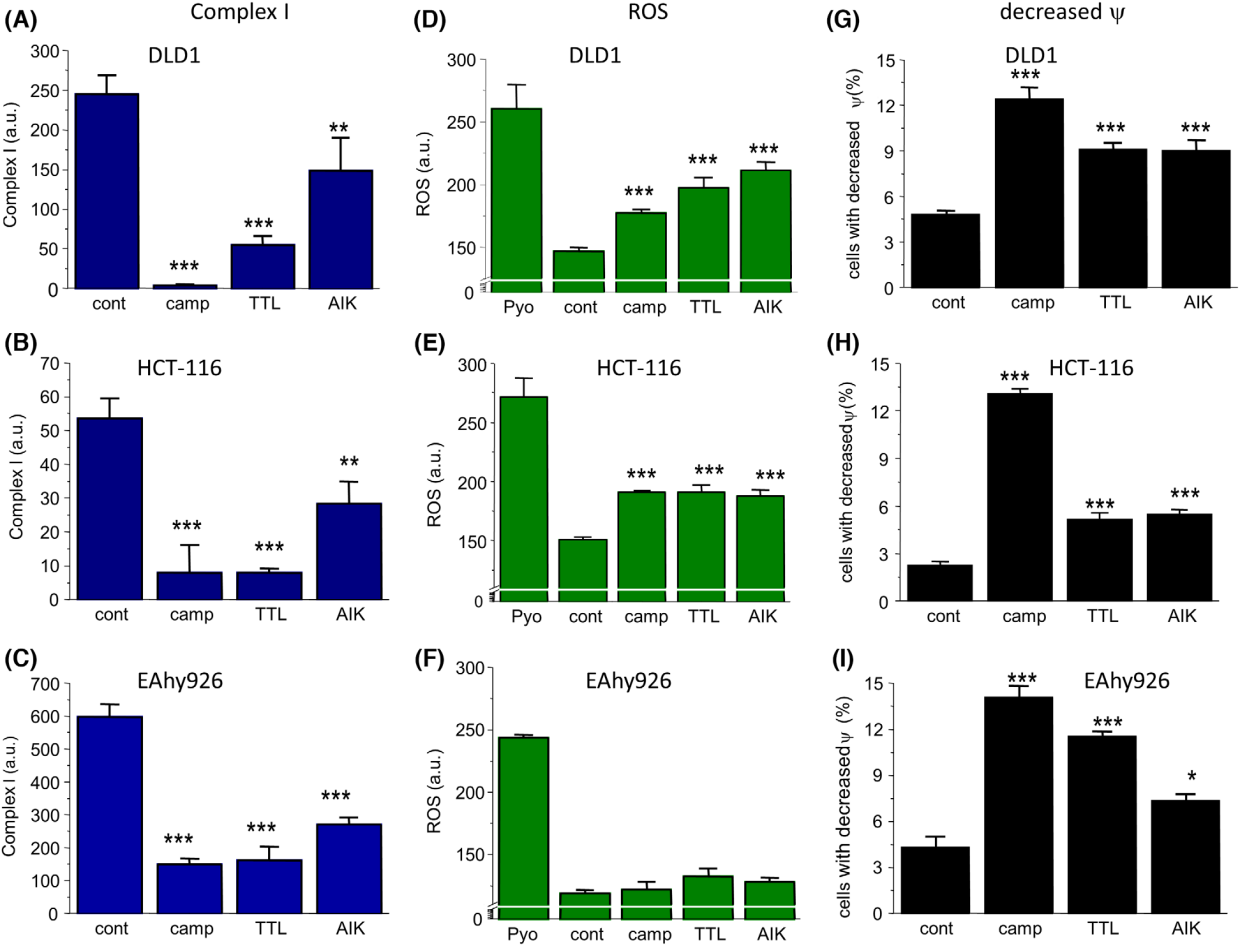
Status of oxidative phosphorylation determined by activity of complex I (A–C), ROS production (D–F), and determination number of cells with decreased mitochondrial membrane potential (G–I) in DLD1 and HCT‐116 cells after treatment with camptothecin (camp), triptolide (TTL), and apoptosis inducer kit (AIK). The activity of complex I (A–C) was decreased in all groups treated with chemotherapeutics in all cell types, whereas ROS production (D, E) was significantly elevated in cancer, but not in non‐cancer EAhy926 cells (F). In these experiments, pyocyanine was used as a control. Also, in all cell lines, the number of cells with decreased mitochondrial membrane potential (G–I) was significantly higher compared to untreated controls. The results are shown as the mean ± SEM and *n* for each type of experiments (shown as number of cultivations/parallels in each cultivation) was: complex I determination, *n* = 3/5; ROS, *n* = 5/4; and changes in Ψ, *n* = 5/3. Significant differences among groups were determined by one‐way ANOVA. For multiple comparisons, an adjusted *t*‐test with *P*‐values corrected by the Bonferroni method was used. *P < 0.05, **P < 0.01, and ***P < 0.001 compared to untreated controls.

In DLD1, HCT‐116, and EAhy926 cell lines, the oxidative phosphorylation (determined by the activity of complex I) was impaired in groups treated with chemotherapeutics (Fig. 3A–C) compared to untreated controls, which was accompanied by increased ROS production (Fig. 3D,E) in DLD1 and HCT‐116 cells, but not in the EAhy926 cell line (Fig. 3F). In these experiments, pyocyanine as a generator of ROS was used as a positive control. An increased number of cells with a decreased mitochondrial membrane potential was determined in all cell lines after the treatment with chemotherapeutics (Fig. 3G–I).

### Size of mitochondria after treatment with chemotherapeutics

**Fig. 4:**
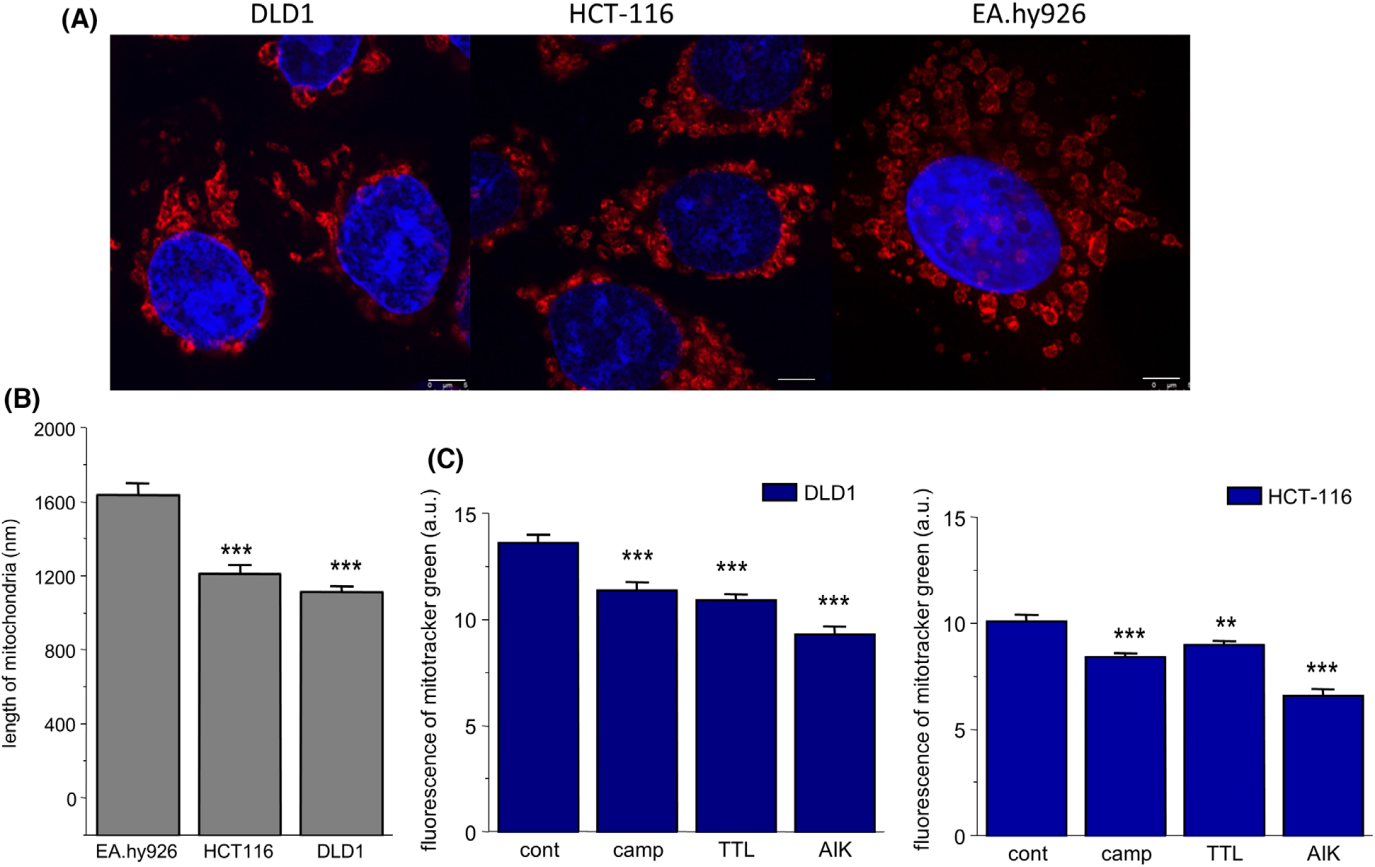
Size of mitochondria in control and chemotherapeutics‐treated groups. As chemotherapeutics, camptothecin (camp), triptolide (TTL), and apoptosis inducer kit (AIK) were used. As determined by confocal microscopy with MitoTracker Red CMXRos, mitochondria were significantly larger in non‐cancer EAhy926 cells compared to cancer DLD1 and HCT‐116 cells (A, B). In all treated groups, a decrease in size of the mitochondria was observed, as determined using a fluorescence reader and MitoTracker Green FM (C). The results are shown as the mean ± SEM and represent an average of three independent cultivations, each performed with five parallels. Significant differences among groups were determined by one‐way ANOVA. For multiple comparisons, an adjusted *t*‐test with *P*‐values corrected by the Bonferroni method was used. **P < 0.01 and ***P < 0.001 compared to untreated controls. Scale bar = 5 μm.

Furthermore, we compared the size of mitochondria in untreated and treated DLD1 and HCT‐116 cells (Fig. 4). In non‐cancer cell line EAhy926, mitochondria were larger compared to cancer DLD1 and HCT‐116 cell lines (Fig. 4A,B). In both these cancer cell lines, we observed a decrease in size of mitochondria determined indirectly by the fluorescence of MitoTracker Green FM after treatment with chemotherapeutics (Fig. 4C). However, from these experiments, it cannot be concluded whether the decreased size was a result of the process of fission, or simply the fragmentation of mitochondria.

### Involvement of fission in the apoptosis induction in HCT‐116 and DLD1 cells after silencing Drp1 and determination of Drp1 expression

**Fig. 5:**
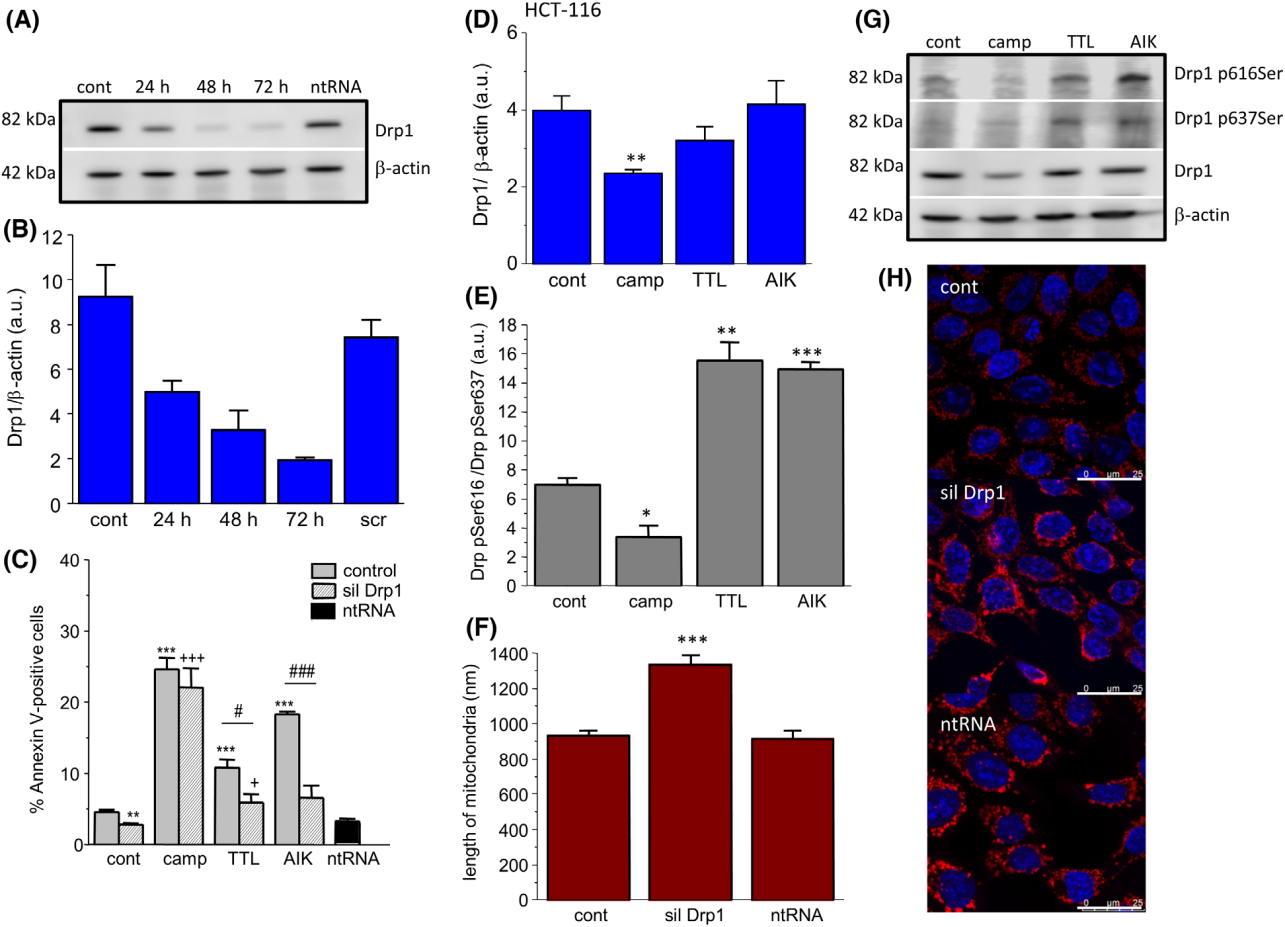
Involvement of fission in the apoptosis induction in HCT‐116 group. Drp1 protein was silenced (siDRP1), and apoptosis was determined in groups treated with camptothecin (camp), triptolide (TTL) and apoptosis inducer kit (AIK). Effectivity of DRP1 silencing was determined after 24, 48, and 72 h (A, B). For experiments, silencing for 48 h was chosen. We observed significant differences between non‐silenced and silenced group treated with TTL and AIK (C). Drp1 protein was significantly decreased in the camptothecin‐treated group (camp) and not in any other groups treated with TTL and/or AIK compared to the control group (D, G). The activity of Drp1 was determined by the ratio Drp1 pSer616 and Drp1 pSer637 (E). An increased ratio was observed in TTL‐ and AIK‐treated groups, thus indictating the process of fission. The size of mitochondria after the silencing was determined by confocal microscopy with MitoTracker Red CMXRos (F, H). Mitochondria were significantly larger in the group treated with Drp1 siRNA compared to the control and/or non‐targeting RNA (ntRNA). The results are shown as the mean ± SEM and *n* for each type of experiments (shown as number of cultivations/parallels in each cultivation) was: determination of Drp1 and β‐actin by western blot analysis, *n* = 4/1; Annexin V cytometric assay, *n* = 3/3; changes in phosphorylated Drp1, *n* = 5/2; and size of mitochondria, *n* = 4/15. Significant differences among groups were determined by one‐way ANOVA. For multiple comparisons, an adjusted *t*‐test with *P*‐values corrected by the Bonferroni method was used. *P < 0.05, **P < 0.01, and ***P < 0.001 compared to untreated controls. #*P* < 0.05 and ###*P* < 0.001 compared to the non‐silenced groups treated with the same chemotherapeutics. +*P* < 0.01 and +++*P* < 0.001 compared to silenced control. Scale bar = 25 μm.

**Fig. 6:**
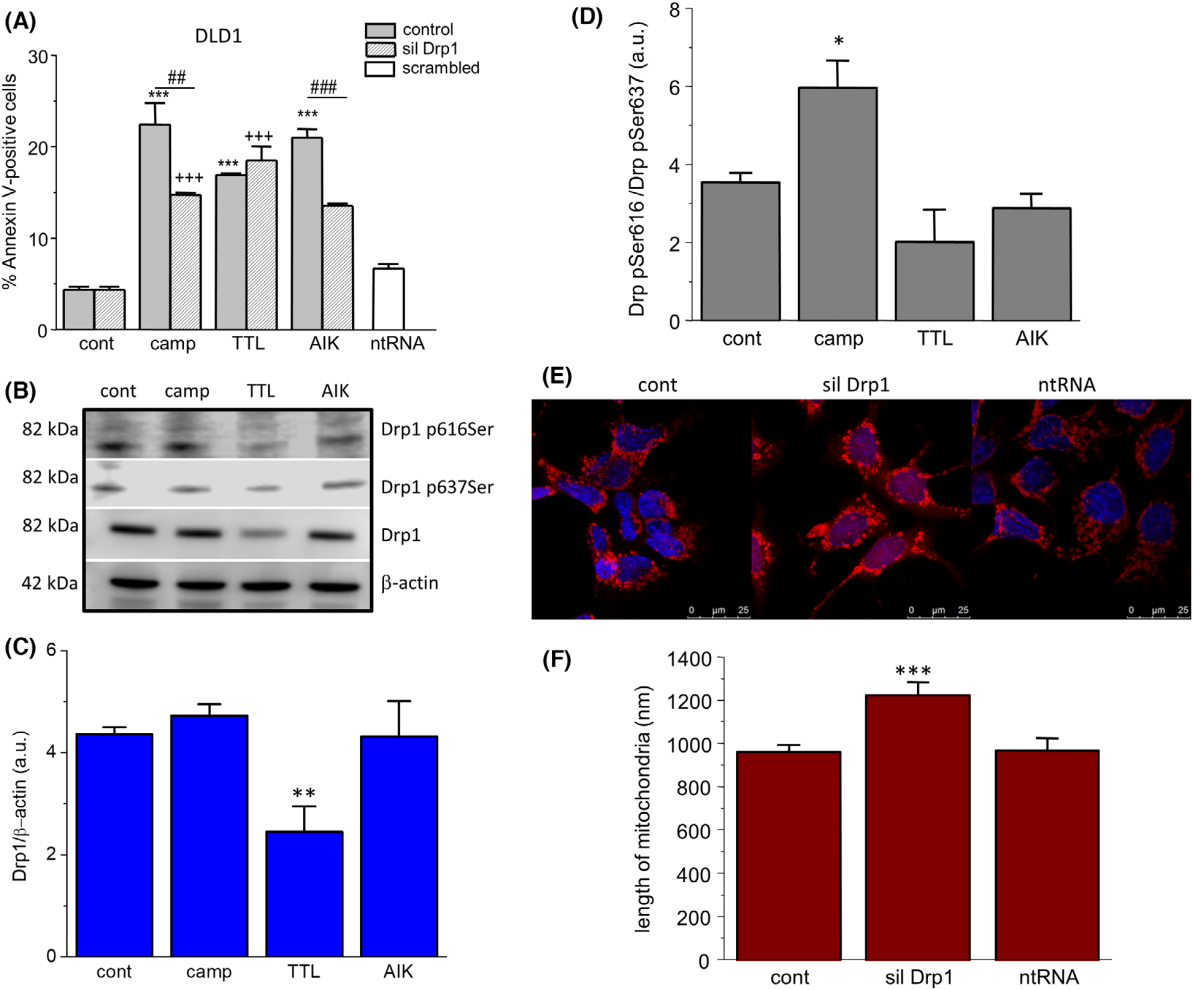
Involvement of fission in the apoptosis induction in the DLD1 group. Drp1 protein was silenced (siDRP1), and apoptosis was determined in groups treated with camptothecin (camp), triptolide (TTL) and apoptosis inducer kit (AIK). By contrast to HCT‐116 cells, in DLD1 cells, we observed significant differences in apoptosis between non‐silenced and silenced groups treated with camptothecin and AIK (A). Drp1 protein was significantly decreased in the TTL‐treated group, and not in any other group treated with camptothecin and/or AIK compared to the control group (B, C). The activity of Drp1 was determined by the ratio Drp1 pSer616 and Drp1 pSer637 (D), showing increased activity in the camptothecin‐treated group compared to control. Comparison of the size of mitochondria (determined by MitoTracker Red CMXRos) revealed a significant increase in the group treated with Drp1 siRNA compared to untreated controls and/or the group treated with non‐targeting siRNA (ntRNA) (E, F). The results are shown as the mean ± SEM and *n* for each type of experiments (shown as number of cultivations/parallels in each cultivation) was: determination of Drp1 and β‐actin by western blot analysis, *n* = 5/1; Annexin V cytometric assay, *n* = 3/3; changes in phosphorylated Drp1, *n* = 3/3; and size of mitochondria, *n* = 4/15. Significant differences among groups were determined by one‐way ANOVA. For multiple comparisons, an adjusted *t*‐test with *P*‐values corrected by the Bonferroni method was used. *P < 0.05, **P < 0.01, and ***P < 0.001 compared to untreated controls. ##*P* < 0.01 and ###*P* < 0.001 compared to the non‐silenced groups treated with the same chemotherapeutics. +++*P* < 0.001 compared to silenced control. Scale bar = 25 μm.

Accordingly, we silenced Drp1, a protein responsible for fission process. Silencing of the Drp1protein was effective for 48 and 72 h compared to non‐silenced control and also compared to control treated with non‐targeting RNA (ntRNA) (Fig. 5A,B). Thus, for other experiments, we used silencing for 48 h. In HCT‐116 cells, we observed that, after treatment with camptothecin, TTL or AIK, apoptosis was lower in the silenced group treated with TTL and AIK compared to treated but non‐silenced groups (Fig. 5C). The effect of silencer on apoptosis was verified by ntRNA. Drp1 protein was significantly decreased in the group of HCT‐116 cells treated with camptothecin (Fig. 5D,G). Furthermore, we determined p616Ser Drp1 and p637Ser Drp1 in control and treated groups. In HCT‐116 cells, Drp1 p616Ser was increased in the group of cells treated with TTL and AIK, but not in the camptothecin‐treated group, and thus the ratio Drp1 p616Ser/Drp1 p637Ser was significantly higher in the TTL and AIK groups (Fig. 5E,G), which suggests mitochondrial fission as a result of these chemotherapeutics. By contrast, in the group of HCT‐116 cells treated with camptothecin, Drp1 p616Ser/Drp1 p637Ser ratio was decreased compared to the control group (Fig. 5E,G). When HCT‐116 cells were treated with Drp1 siRNA, the size of mitochondria was significantly increased compared to the control and ntRNA‐treated groups (Fig. 5F,H). In DLD1 cells, silencing of Drp1 revealed a decrease in apoptosis in camptothecin and AIK‐treated cells compared to the group treated with ntRNA (Fig. 6A). Surprisingly, silencing of Drp1 and subsequent treatment with TTL did not change apoptosis induction (Fig. 6A). Drp1 protein was significantly decreased in the group of DLD1 cells treated with TTL (Fig. 6B,C). The ratio Drp1 p616Ser/Drp1 p637Ser was elevated in the camptothecin‐treated group, but not the changed TTL and AIK groups (Fig. 6B,D). Also in DLD1 cells, treatment with Drp1 siRNA significantly increased the size of mitochondria compared to the control and ntRNA‐treated groups (Fig. 6E,F).

## Discussion

The effectivity of chemotherapy is a major determinant of success in the treatment of tumors. Different chemotherapeutics target various processes in the cells with the aim of inducing apoptosis. We have shown that, in both colorectal carcinoma cell lines (DLD1 and HCT‐116), camptothecin, triptolide, and AIK were able to increase apoptosis and they significantly decreased viability even after 24 h of treatment. The effectivity of these chemotherapeutics on colorectal carcinoma cell lines was supported by decreased ATP production and decreased levels of pyruvate. These chemotherapeutic compounds have different mechanisms of action, with camptothecin being a potent inhibitor of topoisomerases. Camptothecin derivatives could inhibit colorectal cancer proliferation via induction of cell cycle arrest and apoptosis *in vitro* and *in vivo* [17]. Triptolide targets several metabolic points, such as ATP‐dependent helicase XBP [18] and nuclear factor‐kappaB inhibitor [12, 19]. AIK is a mixture of five chemotherapeutics. Actinomycin D belongs to the group of antineoplastic antibiotics, which are known to inhibit RNA synthesis. Camptothecin functions as an inhibitor of nuclear topoisomerase and was shown to induce apoptosis in variety of cell types. Another chemotherapeutics that inhibits translation and thus results in cell growth arrest and cell death in eukaryotes is cycloheximide. Dexamethasone belongs to a group of glucocorticoids. It probably induces apoptosis by binding to the intracellular glucocorticoid receptor, which activates this receptor. Etoposide, a derivative of podophyllotoxin, inhibits topoisomerase activity [20]. Based on these differences, we investigated how these chemotherapeutics affect mitochondria in two stable colorectal carcinoma cell lines (DLD1 and HCT‐116) and also whether fission could be involved in the process of apoptosis.

Mitochondria are organelles that play an important role not only in energetic metabolism, but also in apoptosis induction. All tested chemotherapeutics suppressed the activity of complex I and thus probably oxidative phosphorylation. Also, an increase in ROS production was observed in both DLD1 and HCT‐116 cell lines after camptothecin, TTL and AIK treatment. ROS are implicated in almost all mitochondrial functions, from ATP generation, [Ca^2+^] buffering to induction of apoptosis. Although low‐to‐moderate levels of ROS are physiological and can be beneficial or necessary for cell survival, high ROS levels are detrimental and are associated with cell death [21]. In our experiments, increased levels of ROS correlate with increased apoptosis in all groups. Interestingly, such an increase was not detectable in the non‐tumor EAHy926 cell line, thus pointing against apoptosis induction. Although the chemotherapeutics used in our experiments also affects non‐cancer EAhy926 cells, the viability of these cells remained much higher after treatment compared to cancer cells. Differences in the targeting of the cancer and non‐cancer cells by chemotherapeutics might be a result of a divergence in some of the metabolic and signaling pathways, which are under detailed investigations (e.g. energy demands, proliferation, calcium signaling, and production of ROS). Indeed, in the ovarian cancer cell line, it has already been shown that increasing mitochondrial ROS by inhibition or knockdown of the ROS‐protective uncoupling protein UCP2 enhances cisplatin‐induced apoptosis [10]. In colorectal carcinoma cells, it was shown that a loss of putative oncogene DJ‐1 resulted in mitochondrial dysfunction and ROS accumulation, thus leading to colorectal carcinoma growth inhibition [22]. Mitochondria, comprising small, but important intracellular organelles, constantly change their morphology according to the requirements of the cell, especially energetic demands [23]. Fission and fusion are two crucial processes that can change both the morphology and function of mitochondria.

It has already been shown that a decreased size of mitochondria corresponds to increased apoptosis and an increased number of cells with low mitochondrial membrane potential [24]. Mitochondrial membrane potential, which reflects mitochondrial functional status, is assumed to maintain the respiratory chain to generate ATP, and a decrease in mitochondrial membrane potential accelerates cellular depletion of ATP, followed by the cytochrome *c* release. All of the chemotherapeutics that were investigated resulted in an increased number of cells with decreased mitochondrial membrane potential, which is in line with depleted ATP levels. Promoting mitochondrial fission enhances the therapeutic efficiency of interleukin 2‐mediated SW480 colorectal cancer cell apoptosis by tanshinone IIA, a chemical compound with cytotoxic and antioxidative effects [25], thus supporting the concept of fission process induced by chemotherapeutics. Nevertheless, although it is clear that fission occurred in cancerous cells, it is difficult to conclude about further fission or fragmentation as a result of chemotherapeutic treatment just from the size of mitochondria. A drop in mitochondrial membrane potential and the expression level of Drp1 could be considered as an indicator of mitochondria fission. Therefore, we determined levels of Drp1 protein, as well as Drp1 p616Ser and Drp1 p637Ser. Phosphorylation of Drp1 is the most studied post‐translational modification that regulates mitochondrial fission. Phosphorylation occurs at two serine residues: S616 and S637 [26]. Drp1‐S616 phosphorylation is cell cycle‐regulated via Cdk1/cyclin B and causes mitochondrial fragmentation [27]. Drp1‐S637 is phosphorylated by the cAMP‐dependent protein kinase A, which likely inhibits mitochondrial fission by impairing Drp1 GTPase activity and preventing translocation of Drp1 to mitochondria [28]. The ratio of intensities of these two phosphoproteins revealed that, in HCT‐116 cells, TTL and AIK treatment (but not camptothecin) caused fission of mitochondria. Interestingly, in the DLD1 cell line, changes in the ratio of both phosphorylated serines were detected after treatment with camptothecin, but not TTL and AIK. These results highlight importance of fission in the chemotherapeutic‐induced apoptosis in both colorectal carcinoma cell lines. Also, a link between Drp1 and the size of mitochondria was shown by confocal microscopy, where the group with silenced Drp1 had significantly larger mitochondria compared to the non‐treated control and/or the group treated with nontargeted RNA in HCT‐116 and DLD1 cells. The observed differences might be accounted for by differences in DLD1 and HCT‐116 cells, probably as a result of the different properties of these cell lines (DLD1 is a human colorectal adenocarcinoma cell line and HCT‐116 is a human colon cancer cell line). HCT‐116 is known to be a highly aggressive cell line with an epithelial morphology and little or no capacity to differentiate. This cell line provides a tool for dissecting the molecular mechanism involved in the metastatic cascade [29]. By contrast to this line, the metastatic potential of DLD1 cells is minimal [30].

Furthermore, to evaluate the role of Drp1 protein in the process of apoptosis, we silenced Drp1 for 48 h, subsequently treated individual groups with chemotherapeutics, and then determined changes in apoptosis induction compared to the non‐silenced group. Silencing of Drp1 resulted in smaller apoptosis in HCT‐116 cells after TTL and AIK treatment, whereas, in DLD1 cells, decreased apoptosis was observed in camptothecin and AIK‐treated cells compared to the non‐silenced but treated groups. Our results support the observation that the fission process has an impact on apoptosis induction.

## Conclusions

In summary, we have shown that, despite different mechanisms of action, camptothecin, TTL, and AIK target mitochondria through the process of fission and affect the induction of apoptosis in DLD1 and HCT‐116 cells, although some differences occurred between these two types.

## Conflicts of interest

The authors declare that they have no conflicts of interest.

## Author contributions

OK contributed to study conceptualization and funding acquisition. VL, MK, and BCH contributed to the methodology. LR contributed to microscopy. OK and VL contributed to writing and preparing the original draft. BCH and LR contributed to writing, reviewing and editing. All authors have read and approved the final version of the manuscript submitted for publication.

## Data Availability

The data that support the findings of the present study are available from the corresponding author upon reasonable request.
